# Electronic and Magnetic Properties of Fluorinated Transition Metal Dichalcogenide 1T-MX_2_F_2_ (X = S, Se, Te) Monolayers

**DOI:** 10.3390/nano16040256

**Published:** 2026-02-15

**Authors:** Lixia Zheng, Chenzhi Liu, Yunfei Gao, Aolin Li, Haiming Duan, Fangping Ouyang

**Affiliations:** 1 School of Materials Science and Engineering, Xinjiang University, Urumqi 830046, China; zhlixia2023@163.com; 2 Xinjiang Key Laboratory of Solid-State Physics and Devices, School of Physics and Technology, Xinjiang University, Urumqi 830046, China; 107552300793@stu.xju.edu.cn (C.L.); k2389772573@163.com (Y.G.); 3 School of Physics, Institute of Quantum Physics, Central South University, Changsha 410083, China

**Keywords:** DFT calculation, two-dimensional materials, surface fluorination

## Abstract

Two-dimensional transition metal dichalcogenides (TMDCs) have attracted worldwide attention due to their rich physical and chemical properties. How to regulate their electronic structures to meet different application requirements is a crucial issue. In this work, based on first-principle calculations, we demonstrate that surface fluorination can be a powerful method for tailoring the electronic and magnetic properties of TMDC monolayers. The fluorinated T-MX_2_F_2_ (X = S, Se, Te) monolayers cover semiconductors, half-metals, semimetals, and half-semimetals. In particular, monolayer T-CrS_2_F_2_ is a half-semimetal, and the spin–orbit coupling effect changes it to a quantum anomalous Hall insulator. Monolayer T-HfS_2_F_2_ is a non-magnetic semimetal, and monolayer T-CoS_2_F_2_ is a half-metal. These findings not only suggest that fluorination can dramatically alter the electronic properties of two-dimensional TMDCs but also provide a new research platform for developing nanoelectronic devices.

## 1. Introduction

In the past decade, two-dimensional (2D) transition metal dichalcogenides (TMDCs) have attracted widespread attention and emerged as a research hotspot in materials science and nanotechnology, due to their excellent physical and chemical properties. They exhibit high carrier mobility [1,2,3,4], excellent thermal conductivity [5], outstanding catalytic activity [6], great sensing performance [7,8], fine biocompatibility [9,10], etc. [11,12,13,14]. Especially in nanoelectronics, 2D TMDCs provide an important research platform for developing the post-Moore electronics utilizing the charge, spin, and valley degrees of the freedom of electron carriers [15]. For example, the fabricated MoS_2_-based field-effect transistors with a gate length of 1 nm have achieved a current on/off ratio as high as 10^5^ times [16]. The HCP-Co/TMDCs magnetic tunnel junction can achieve a tunneling magnetoresistance (TMR) of up to 3600% [17]. The built-in electric field of the Janus monolayer CrSSe can induce valley polarization up to 71 meV [18].

Although 2D TMDCs cover a lot of materials, some special materials are still rare in this family, such as semimetals [19], magnetic semiconductors [20], half-metals [21], and topological insulators [22]. How to tailor their electronic properties to meet diverse application requirements is still a crucial issue. Commonly used strategies include chemical doping [23], defects, strain, gating, stacking, and alloying [24,25]. For example, the application of biaxial or uniaxial tensile strain (0–10%) can control the electronic and magnetic properties of a series of early-transition metal dichalcogenide monolayers [26]. Despite the strong regulatory effect, disordered defects, doping, and alloying will inevitably lead to a decrease in carrier mobility and performance stability. It is difficult to maintain large strains in two-dimensional materials due to weak van der Waals interactions. Exploring new regulatory methods is of great significance for expanding the applications of 2D TMDCs. Notably, based on first-principle calculations, Li et al. found that surface fluorination can change monolayer H-MoS_2_, a non-magnetic semiconductor, into half-semimetallic T-MoS_2_F_2_ with room-temperature ferromagnetism [27]. Meanwhile, fluorine is the element with the highest electronegativity, forming strong bonds with surface atoms and exhibiting excellent environmental stability. This suggests that fluorination may be a powerful method for regulating the electronic and magnetic properties of two-dimensional materials [27]. But so far, few studies have been conducted on the surface fluorination effect of 2D TMDCs.

In this work, we investigated the electronic and magnetic properties of surface-fluorinated TMDC monolayers T-MX_2_F_2_ (X = S, Se, Te). Based on first-principle calculations, 15 stable T-MX_2_F_2_ (X = S, Se, Te) monolayers were filtered out, covering semiconductors, metals, semimetals, and half-semimetals. Representatively, the electronic properties of monolayers CrS_2_F_2_, HfS_2_F_2_, CoS_2_F_2_, and FeS_2_F_2_ are discussed to demonstrate fluorination effects.

## 2. Calculation Details

In this work, all first-principle calculations based on density functional theory (DFT) are carried out using the Vienna Ab-initio Simulation Package (VASP 5.4.4) [28,29]. The electron–core interactions are described with the projector augmented-wave method (PAW) [30]. The plane wave cutoff energy was taken as 500 eV. The Perdew–Burke–Ernzerhof (PBE) functional was used to describe the exchange-correlation potential [31]. If not specifically mentioned, Hubbard U correction was not adopted for transition metal atoms, because standard DFT calculations give results consistent with those obtained using HSE06 hybrid functionals (see Figure S12 for details). A vacuum layer thickness of 20 Å was used to eliminate the fake interlayer interactions induced by the periodic boundary conditions. In the structural optimization calculations, the convergence criteria of total energy and force were set as 10^−6^ eV and 0.01 eV/Å, respectively. A 16 × 16 × 1 Γ-centered Monkhorst–Pack grid was used to sample first Brillouin zone. We employed a 4 × 4 × 1 supercell model and a 4 × 4 × 1 k-point mesh for phonon spectrum calculations. The ab initio molecular dynamics (AIMD) simulation was performed at 500 K on the 4 × 4 × 1 supercell with a time step of 1.5 fs for a total duration of 10 ps. To estimate the Curie temperatures, Monte Carlo simulations were performed based on the Heisenberg model using the MCsolver code. In addition, Wannier90, VASPberry and WannierTools codes were used to analyze the topological properties. The Chern number *C* is calculated by integrating the Berry curvature of all valence bands over the Brillouin zone:

(1) $$C=\sum_{n}\frac{1}{2\pi }\int_{BZ}\Omega_{xy}^{n}(k)dk_{x}dk_{y}$$

And the anomalous Hall conductance *σ_xy_* is calculated with the Kubo formula:

(2) $$\sigma_{xy}=\frac{e^{2}}{\hbar }\sum_{n}\int_{\mathrm{BZ}}\frac{d^{2}k}{{\left(2\pi \right)}^{2}}\Omega_{xy}^{n}f_{n}$$

In Equations (1) and (2), *n*, Ω, and *f_n_* are the band index, Berry curvature, and Fermi-Dirac distribution function, respectively.

To evaluate the feasibility of fluorinated TMDCs, the fluorination formation energy of T-MX_2_F_2_, *E_f_*, is defined based on the following equation:

(3) $$E_{f}=E_{MX_{2}F_{2}}-E_{MX_{2}}-2\mu_{F}$$

where *E_MX_*_2*F*2_, *E_MX_*_2_ and *μ_F_* represent the total energies of T-MX_2_F_2_, T-MX_2_, and the chemical potential of *F* in *F*_2_ gas, respectively. Thereby, a negative value indicates the surface fluorination process is a spontaneous reaction.

## 3. Results and Discussion

**Figure 1:**
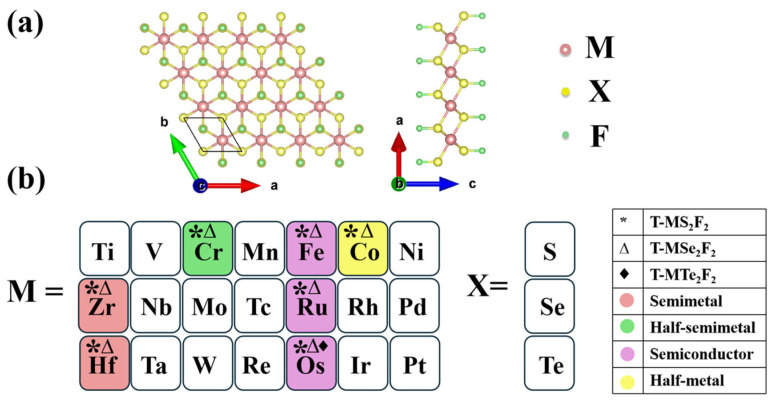
(**a**) Crystal structure of surface-fluorinated T-MX_2_F_2_ monolayers. (**b**) Schematic of stable T-MX_2_F_2_ monolayers. White indicates that the structure is unstable, while other colors indicate the structure is dynamically and thermodynamically stable. Symbols asterisk (*), triangle (∆), and rhombus (♦) represent X = S, Se, Te, respectively. Colors red, green, purple, and yellow represent semimetal, half-semimetal, semiconductor, and metal, respectively.

As shown in Figure 1a, the surface-fluorinated T-MX_2_F_2_ adopted a five-atom-layer stacked configuration of F–X–M–X–F, where X represents S, Se, and Te, and M represents transition metal atoms in groups IVB–VIII. The DFT results suggest that the top of the X atoms is the most stable adsorption site for F. A total of 63 T-MX_2_F_2_ monolayers was calculated. To evaluate their stability, we carried out AIMD simulations and phonon spectrum calculations. Fifteen stable T-MX_2_F_2_ monolayers were screened out, as colored in Figure 1b. We use asterisk (*), triangle (∆), and rhombus (♦) symbols to represent T-MS_2_F_2_, T-MSe_2_F_2_, and T-MTe_2_F_2_, respectively. For instance, Cr^*Δ^ indicates that monolayer T-CrS_2_F_2_ and T-CrSe_2_F_2_ are stable, but monolayer T-CrTe_2_F_2_ is unstable. In addition, these fluorinated monolayers exhibit diverse electronic properties, covering semiconductors, half-metals, semimetals, and half-semimetals. We use different colors in Figure 1b to distinguish these properties. Representatively, we selected T-CrS_2_F_2_, T-HfS_2_F_2_, T-CoS_2_F_2_, and T-FeS_2_F_2_ as case studies to discuss the electronic and magnetic properties of T-MX_2_F_2_ monolayers in the following sections. The energy bands and stability of other materials are detailed in Supplementary Materials Figures S1–S6.

### 3.1. Half-Semimetallic T-CrS_2_F_2_

**Figure 2:**
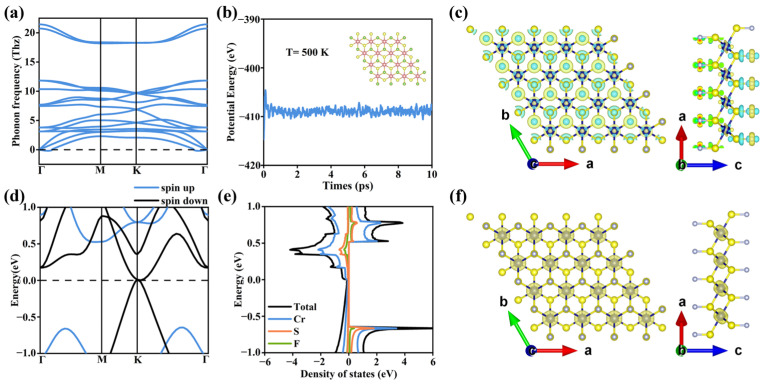
(**a**) The phonon spectrum of monolayer T-CrS_2_F_2_. (**b**) The ab initio molecular dynamics simulation results at 500 K. (**c**) The differential charge density; yellow and cyan represent electron accumulation and loss, respectively. (**d**) The band structure of monolayer T-CrS_2_F_2_ without considering SOC effects. (**e**) The TDOS and PDOS for each element. (**f**) The spin density distribution.

By comparing the total energies of different magnetic orders (see Figure S19 and Table S5 for details), we find that monolayer T-CrS_2_F_2_ has the ferromagnetic (FM) ground state. Figure 2a shows the phonon dispersion of monolayer T-CrS_2_F_2_ in the FM ground state. Except for a few negligibly small imaginary frequencies near the Γ point, there is no imaginary frequency over the Brillouin zone, suggesting monolayer T-CrS_2_F_2_ is dynamically stable. Figure 2b shows the total energy evolution of monolayer T-CrS_2_F_2_ in the AIMD simulation under 500 K, which remains stable throughout the entire simulation interval. The embedded image in Figure 2b displays the final structure of T-CrS_2_F_2_. There is no obvious structural distortion, suggesting monolayer T-CrS_2_F_2_ is thermodynamically stable. In addition, the fluorination formation energy *E_f_* for T-CrS_2_F_2_ is −3.02 eV per formula unit (eV/f.u.), indicating strong chemical bonding between F and S atoms, and monolayer T-CrS_2_F_2_ may be experimentally achievable.

Interestingly, contrary to conventional expectations that fluorination will change the transition metal atom Cr from the Cr^4+^ state for CrS_2_ into the Cr^6+^ state for T-CrS_2_F_2_, our differential charge density analysis in Figure 2c suggests that the charge transfer after fluorination mainly occurs between S and F atoms, while the charges on Cr atoms change much less. As a result, the Cr atom remains in the Cr^4+^ state in monolayer T-CrS_2_F_2,_ and the 3d^2^4s^0^ valence state causes 2 μ_B_ net magnetic moments per Cr^4+^ ion. Consistently, our DFT calculations confirm that monolayer T-CrS_2_F_2_ has 2 μ_B_ net magnetic moments per unit cell, and the local magnetic moments are mainly contributed by Cr atoms, as shown by the spin density distribution in Figure 2f.

Figure 2d displays the band structure of monolayer T-CrS_2_F_2_ without considering the spin–orbit coupling (SOC) effect. The spin-up bands are gapped, while the spin-down bands pass through the Fermi level, suggesting T-CrS_2_F_2_ is half-metallic. For the spin-down bands, there is only a Weyl point located at high-symmetry point K, resulting in zero total density of states (TDOS) at the Fermi level in Figure 2e, indicating a semimetal nature. Overall, the DFT results suggest monolayer T-CrS_2_F_2_ is an intrinsic 2D half-semimetal. We also calculated the band structure using the HSE06 hybrid functional, which gives consistent results (see Figure S12 in Supplementary Materials).

**Figure 3:**
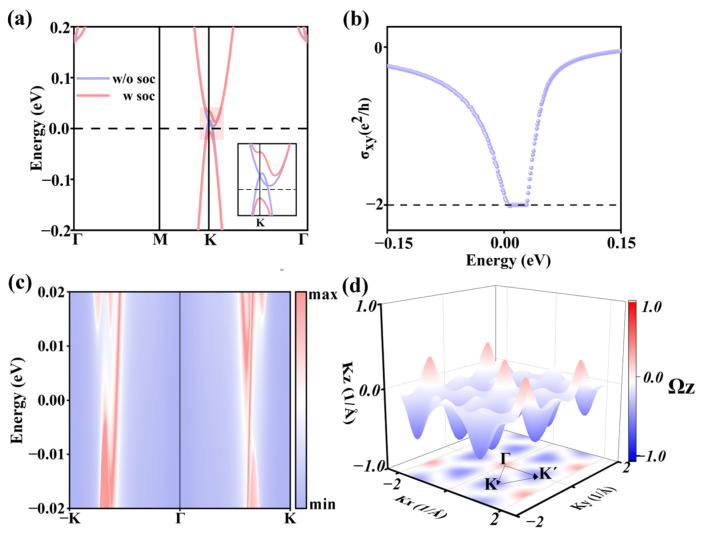
(**a**) Band structures of monolayer T-CrS_2_F_2_ near the Fermi level, considered without (w/o) and with (w) the SOC effects. (**b**) The abnormal Hall conductivity of monolayer T-CrS_2_F_2_. (**c**) The topological edge states. (**d**) Berry curvature of the valence bands.

Interestingly, after considering the SOC effect, a band gap of 21.6 meV opens at the Weyl point for monolayer T-CrS_2_F_2_, as shown in Figure 3a. This indicates monolayer T-CrS_2_F_2_ may be a topological insulator. To verify this, we calculated the Chern number of monolayer T-CrS_2_F_2_. By integrating the Berry curvatures of the valence bands over the whole Brillouin zone, as displayed in Figure 3d, we obtained a non-zero Chern number *C* = −2, confirming monolayer T-CrS_2_F_2_ is a quantum anomalous Hall insulator (QAHI). Using DFT and Maximally localized Wannier functions (MLWFs) (see Figure S7a for details), we further calculated the topological surface states. As shown in Figure 3c, two linearly dispersing states connect the valence and conduction bands of monolayer T-CrS_2_F_2_, corresponding to the two topologically protected chiral edge channels. In Figure 3b, the quantized Hall conductivity of *σ_xy_* = −2e^2^/h and the width of *σ_xy_* plateau Δ*E* = 16 meV is in line with the calculated Chern number *C* = −2 and the energy gap *E*_g_ = 22 meV, respectively. The small difference between Δ*E* and *E*_g_ is caused by the broadening of the Fermi–Dirac distribution function in the calculation.

The estimated Curie temperature given by the Monte Carlo simulation is 577 K for monolayer T-CrS_2_F_2_ in Figure S9. The half-metallicity and novel electronic topological properties make monolayer T-CrS_2_F_2_ a potential platform for developing low-power, high-speed nanologic devices and non-volatile memory.

**Figure 4:**
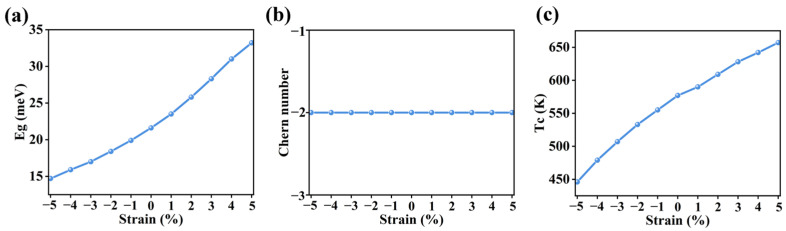
(**a**) The energy gap of monolayer T-CrS_2_F_2_ considering the SOC effect, (**b**) the Chern number, and (**c**) the Curie temperature as a function of strain *ε*.

The strain effects on the electronic and magnetic properties of monolayer T-CrS_2_F_2_ are also investigated. The strength of biaxial strain (*ε*) is defined as the change rate of the in-plane lattice constant:

(4) $$\varepsilon =\left(a-a_{0}\right)/a_{0}\times 100\%$$

where *a*_0_ and *a* are the in-plane lattice constant without and with strain, respectively. Positive and negative ε values represent tensile and compressive strain, respectively. As displayed in Figure 4a,b, the band gap monotonically increases with the increase in strain, while the Chern number remains unchanged, suggesting the topological properties of monolayer T-CrS_2_F_2_ is robust against strain. Figure 4c suggests that increasing strain will lead to a decrease in Curie temperature. But in the studied strain range, the Curie temperature is always higher than room temperature.

### 3.2. Semimetallic T-HfS_2_F_2_

**Figure 5:**
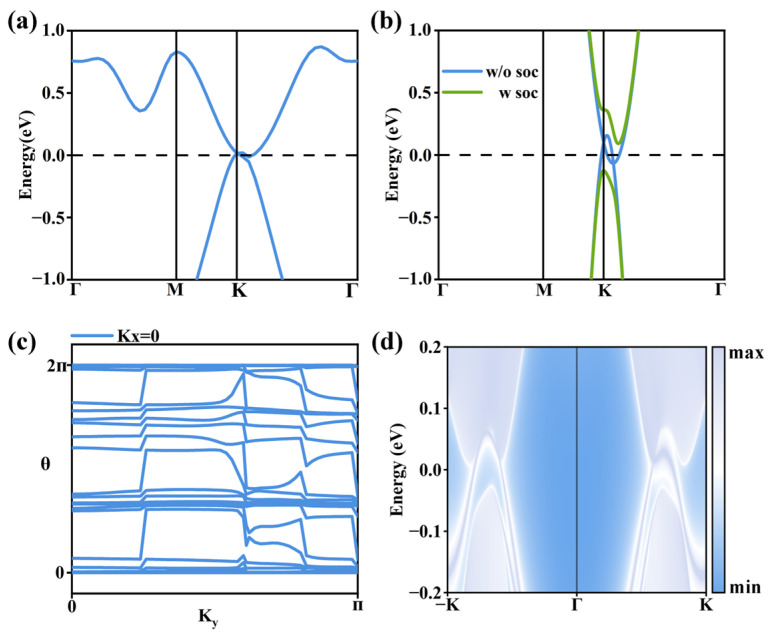
(**a**) Band structure of monolayer T-HfS_2_F_2_ without considering SOC effects. (**b**) Band structures of monolayer T-HfS_2_F_2_ near the Fermi level, without (w/o) and with (w) the consideration of SOC effects. (**c**) The Wilson loop spectrum of monolayer T-HfS_2_F_2_. (**d**) The edge states of monolayer T-HfS_2_F_2_.

The band structure of monolayer T-HfS_2_F_2_ is displayed in Figure 5a. All bands are spin-degenerate, and the DFT calculations give zero total magnetic moments per unit cell, indicating monolayer T-HfS_2_F_2_ is non-magnetic. Similarly to T-CrF_2_S_2_, in T-HfX_2_S_2_, the charge transfer after fluorination mainly occurs between the S and F atoms, while the charge on Hf atoms changes much less, as shown in Figure S20b. Therefore, in monolayer T-HfS_2_F_2_, the Hf atoms remain in the Hf^4+^ state, achieving a valence state of 5d^0^6s^0^. Also, similarly to monolayer T-CrS_2_F_2_, the Weyl point at the Fermi level makes monolayer T-HfS_2_F_2_ an intrinsic semimetal. In Figure 5b, a band gap of 49.1 meV opens after considering SOC effects. To verify whether it is a topological insulator, based on DFT and MLWFs calculations (see Figure S7b for details), we calculate the edge states of monolayer T-HfS_2_F_2_ in Figure 5d. Obviously, some edge states cross over the energy gap. However, the Wilson loop spectrum in Figure 5c gives a result of Z2 = 0 since the curves always cross a line parallel to the k*_y_*-axis an even number of times. This result indicates that the metallic edge states may change into gapped via edge passivation or other treatments due to the trivial topological properties.

### 3.3. Half-Metallic T-CoS_2_F_2_

**Figure 6:**
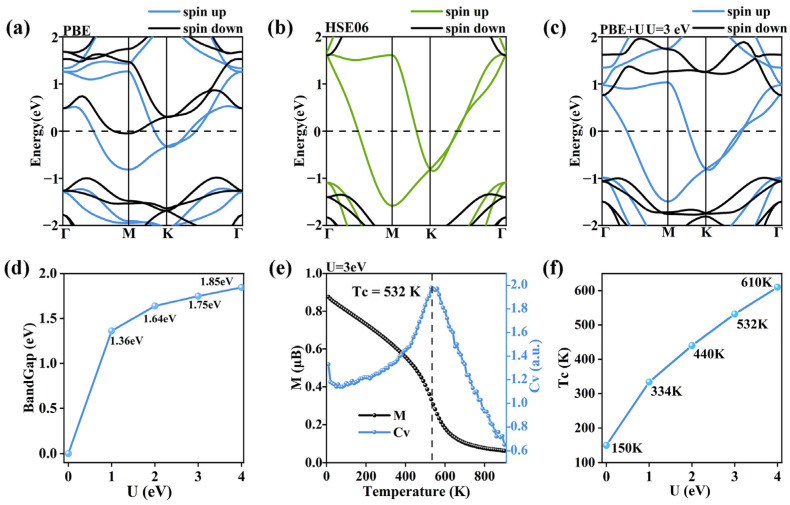
(**a**–**c**) The band structure of monolayer T-CoS_2_F_2_ calculated using the (**a**) standard DFT method, (**b**) HSE06 hybrid functional method, and (**c**) DFT+U method (U = 3 eV). (**d**) The band gap as a function of U. (**e**) Monte Carlo simulation results for T-CoS_2_F_2_ using the DFT+U method (U = 3 eV). (**f**) The Curie temperature as a function of the U value.

Standard DFT calculations in Figure 6a show that monolayer T-CoS_2_F_2_ is a magnetic metal. However, the HSE06 hybrid functional calculations in Figure 6b suggest monolayer T-CoS_2_F_2_ should be a half-metal. Considering that the standard DFT calculations usually underestimate the electron correlation effects in localized 3d orbits, we adopted the Hubbard U correction for monolayer T-CoS_2_F_2_ to address this disagreement. A typical U value for Co-3d orbital is usually about 1–4 eV in previous works. As shown in Figure 6c, when Hubbard U correction with *U*_eff_ = 3 eV is used, only the spin-up band passes through the Fermi level, while the spin-down bands are gapped, which is in reasonable agreement with the HSE06 hybrid functional results. To study the influence of the *U* value on the electronic structure, we calculated the band structure of monolayer T-CoS_2_F_2_ with U = 0 eV, 1 eV, 2 eV, 3 eV, and 4 eV, respectively. In Figure 6d, as the U value increases, the band gap of the spin-down bands increases while the spin-up bands remain metallic, suggesting the half-metallicity of monolayer T-CoS_2_F_2_ is stable against a wide range of U values. Based on Monte Carlo simulations, the Curie temperature of monolayer T-CoS_2_F_2_ is approximately 532 K for U_eff_ = 3 eV, as shown in Figure 6e,f. It increases with an increase in U. Notably, the specific heat diverges toward 0K because monolayer T-CoS_2_F_2_ has easy-plane magnetic anisotropy, which allows the spin orientation to freely rotate along any in-plane orientation under arbitrary small perturbations. The divergence of heat capacity at 0 K indicates that monolayer T-CoS_2_F_2_ is actually in the superparamagnetic state below T_C_.

### 3.4. Semiconductive T-FeS_2_F_2_

**Figure 7:**
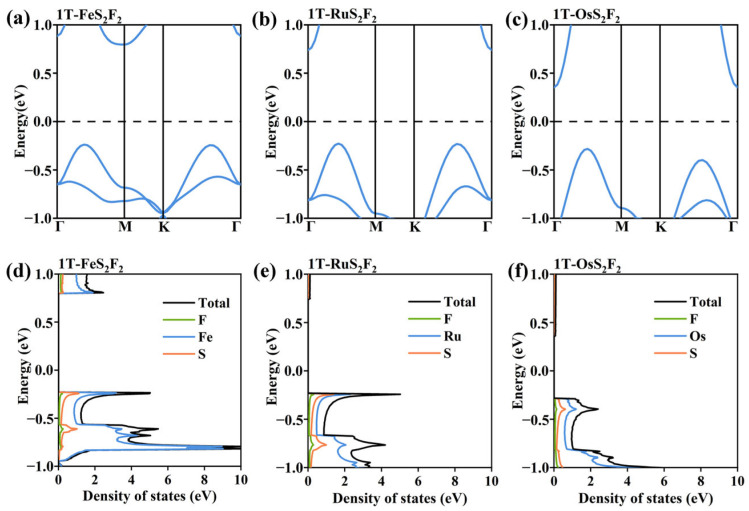
(**a**–**c**) The band structure of (**a**) monolayer T-FeS_2_F_2_, (**b**) monolayer T-RuS_2_F_2_, and (**c**) monolayer T-OsS_2_F_2_. (**d**–**f**) The TDOS and PDOS of each element for (**d**) monolayer T-FeS_2_F_2_, **(e)** monolayer T-RuS_2_F_2_, and (**f**) monolayer T-OsS_2_F_2_.

For monolayer T-FeS_2_F_2_, all bands in Figure 7a are spin-degenerate, and the DFT calculations give zero total magnetic moments per unit cell, indicating monolayer T-FeS_2_F_2_ is a non-magnetic semiconductor. Without considering SOC, the Fermi level does not pass through the valence and conduction bands. Meanwhile, the density of states shows that the electronic density of states near the Fermi level is 0, further proving that monolayer T-FeS_2_F_2_ is a non-magnetic semiconductor. The T-RuS_2_F_2_ and T-OsS_2_F_2_ of the same group exhibit similar characteristics. Their band structures are shown in Figure 7b,c, respectively, and their state densities are shown in Figure 7e,f.

## 4. Summary

In summary, based on first-principle calculations, we study the stability and electronic properties of monolayer-fluorinated transition metal disulfide compounds 1T-MX_2_F_2_ (X = S, Se, Te), covering half-semimetal, semimetal, half-metal, and non-magnetic semiconductors. The results show that monolayer T-CrS_2_F_2_ is a half-semimetal material with nontrivial topological properties, exhibiting quantized Hall conduction and chiral edge states. Monolayer T-HfS_2_F_2_ is a non-magnetic semimetal. Monolayer T-CoS_2_F_2_ exhibits intrinsic half-metallicity, and T-FeS_2_F_2_ is a non-magnetic semiconductor. These findings demonstrate the great potential of two-dimensional magnetic materials in future spintronic device applications.

## Data Availability

The original contributions presented in this study are included in the article/Supplementary Material. Further inquiries can be directed to the corresponding authors.

## Supplementary Materials

The following supporting information can be downloaded at https://www.mdpi.com/article/10.3390/nano16040256/s1: Figure S1. The band structure of monolayer (a) T-ZrS_2_F_2_, (b) T-ZrSe_2_F_2_, (c) T-HfSe_2_F_2_.; The phonon spectrum of monolayer (d) T-ZrS_2_F_2_ (e) T-ZrSe_2_F_2_ (f) T-HfSe_2_F_2_. Figure S2. The band structure of monolayer (a) T-FeSe_2_F_2_, (b) T-RuSe_2_F_2_, (c)T-OsSe_2_F_2_.; The phonon spectrum of monolayer (d) T-FeSe_2_F_2_ (e) T-RuSe_2_F_2_ (f)T-OsSe_2_F_2_. Figure S3. The band structure of monolayer (a) T-RuS_2_F_2_, (b) T-OsS_2_F_2_, (c) T-OsTe_2_F_2_.; The phonon spectrum of monolayer (d) T-RuS_2_F_2_, (e) T-OsS_2_F_2_, (f) T-OsTe_2_F_2_. Figure S4. (a)The band structure of monolayer T-CrSe_2_F_2_. (b)The phonon spectrum of monolayer T-CrSe_2_F_2_. Figure S5. Under U=3 eV, (a) the band structure of monolayer T-CoSe_2_F_2_. and (b) the phonon spectrum of monolayer T-CoSe_2_F_2_. Figure S6. (a) The phonon spectrum of monolayer T-HfS_2_F_2_; (b) The phonon spectrum of monolayer T-CoS_2_F_2_. (c)The phonon spectrum of monolayer T-FeS_2_F_2_. Figure S7. (a) The band structures of monolayer T-CrS_2_F_2_ obtained by DFT and MLWFs calculations. The Fermi level is set as 0 eV. (b) The band structures of monolayer T-HfS_2_F_2_ obtained by DFT and MLWFs calculations. The Fermi level is set as 0 eV. Figure S8. The two magnetic orders (a) FM, (b) AFM. Figure S9. The Curie temperature of T-CrS_2_F_2_ is determined through Monte Carlo simulations. Figure S10. The band structure of monolayer T-CoS_2_F_2_. (a) U = 1 eV, (b) U = 2 eV, (c) U = 3 eV, (d) U = 4 eV. Figure S11. Magnetic moments (*M*) and specific heat (*C*_V_) obtained by MC simulations for T-CoS_2_F_2_ (a) U = 1 eV, (b) U = 2 eV, (c) U = 3 eV, (d) U = 4 eV. Figure S12. Band structure without considering the SOC effect. Using HSE06 hybrid functional (a) T-CrS_2_F_2_, (b) T-CoS_2_F_2_, (c) T-HfS_2_F_2_, and (d) T-FeS_2_F_2_. Using PBE functional (e) T-CrS_2_F_2_, (f) T-CoS_2_F_2_, (g) T-HfS_2_F_2_, and (h) T-FeS_2_F_2_. Figure S13. The band structures of monolayer T-MoS_2_F_2_ under different functionals (a) HSE06 hybrid functional, (b) PBE functional, and (c) PBE+U functional, respectively. Figure S14. The band structure of monolayer T-CrS_2_F_2_ under strain without considering SOC. (a) tensile strain; (b) compressive strain. Figure S15. The band structure of monolayer T-CrS_2_F_2_ under strain considering SOC. (a) tensile strain; (b) compressive strain. Figure S16. Through Monte Carlo simulation, the Curie temperature of monolayer T-CrS_2_F_2_ under strain (a) tensile strain; (b) compressive strain. Figure S17. The band structure of monolayer T-CoS_2_F_2_ under strain. (a) tensile strain; (b) compressive strain. Figure S18. Through Monte Carlo simulation, the Curie temperature of monolayer T-CoS_2_F_2_ under strain (a) tensile strain; (b) compressive strain. Figure S19. Four different magnetic order configurations: (a) FM (b) AFM1 (c) AFM2 (d) AFM3. Figure S20. Differential charge density of (a) monolayer T-CrS_2_F_2_, T-HfS_2_F_2_, T-CoS_2_F_2_, and T-FeS_2_F_2_, respectively. Yellow and cyan represent electron accumulation and loss, respectively. Table S1. The fluorination formation energy *E*_f_ (eV/formula unit) for T-MX_2_F_2_. Table S2. The total energies of two different magnetic order energies in unit of eV/f.u. Table S3. The total energy of the ferromagnetic (E_FM_) and antiferromagnetic (E_AFM_) states of T-CrS_2_F_2_, as well as the nearest-neighbor exchange coupling parameter (*J_1_*) and the magnetic anisotropy parameter (*A_i_*). Table S4. The total energy of the ferromagnetic (E_FM_) and antiferromagnetic (E_AFM_) states of T-CoS_2_F_2_, as well as the nearest-neighbor exchange coupling parameter (*J*_1_) and the magnetic anisotropy parameter (*A_i_*). Table S5. The total energies of four different magnetic order energies in unit of eV/f.u.
