# Supplementary material for: Electronic and Magnetic Properties of Fluorinated Transition Metal Dichalcogenide 1T-MX2F2 (X = S, Se, Te) Monolayers
Source: Nanomaterials (Basel). 2026 Feb 15;16(4):256. doi: 10.3390/nano16040256 (PMC12943228; doi:10.3390/nano16040256)
Supplement: Supplementary file 1 [file nanomaterials-16-00256-s001.zip › nanomaterials-4140629-supplementary.pdf]

Supplementary Material of "Electronic and magnetic properties of fluorinated transition metal dichalcogenide 1T-MX_2F_2 (X=S, Se, Te) monolayers"

Lixia Zheng [1], Chenzhi Liu [2], Yunfei Gao [2], Aolin Li [1, 2*], Haiming Duan [2*], Fangping Ouyang [2,3*]

[1] School of Materials Science and Engineering, Xinjiang University, Urumqi 830046, China

[2] School of Physics and Technology, and Xinjiang Key Laboratory of Solid-State Physics and Devices, Xinjiang University, Urumqi 830046, China

[3] School of Physics, and Institute of Quantum Physics, Central South University, Changsha 410083, China

* Corresponding author emails: liaolin628@xju.edu.cn; dhm@xju.edu.cn; ouyangfp06@tsinghua.org.cn

Part I Electronic structure and phonon spectrum

The band structure of monolayer T-ZrS_2F_2 is displayed in Figure S1(a). All bands are spin-degenerate, and the DFT calculations give zero total magnetic moments per unit cell, indicating monolayer T-ZrS_2F_2 is non-magnetic. The Weyl point at the Fermi level makes monolayer T-HfS_2F_2 an intrinsic semimetal. The phonon dispersion of monolayer T-ZrS_2F_2 is displayed in Figure S1(d). Except for some negligibly small imaginary frequencies near the Γ point due to numerical errors induced by the acoustic sum rule, the absence of imaginary modes throughout the Brillouin zone (BZ) suggests monolayer T-ZrS_2F_2 is dynamically stable. The band structures of T-$ZrSe_2F_2$ and T-$HfSe_2F_2$, as shown in Figures S1(b) and S1(c), and their stabilities, as depicted in Figures S1(e) and S1(f), are similar.

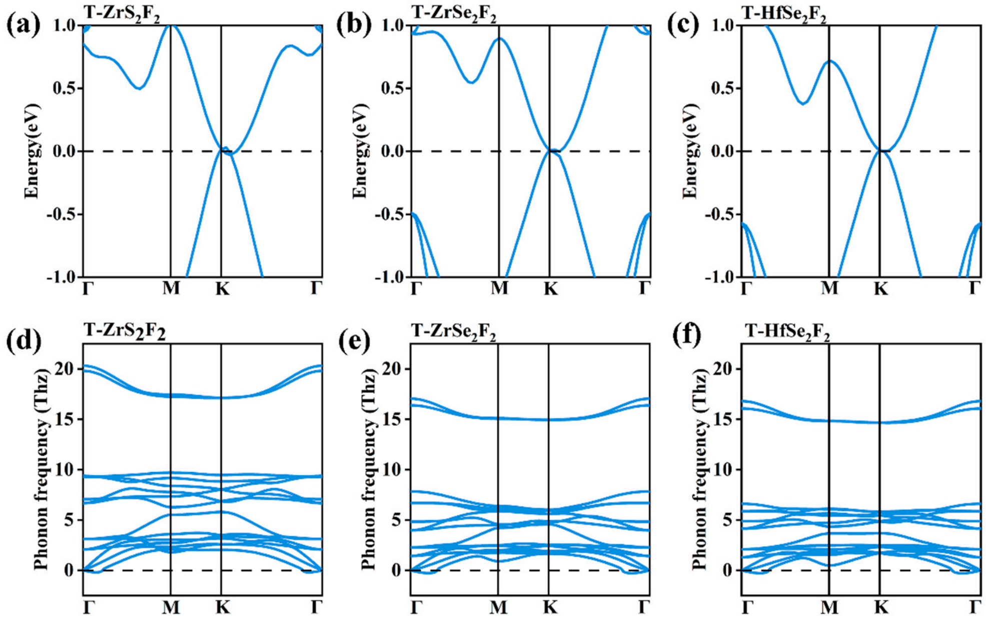

Figure S1. The band structure of monolayer (a) T-ZrS_2F_2, (b) T-$ZrSe_2F_2$, (c) T-$HfSe_2F_2$.; The phonon spectrum of monolayer (d) T-ZrS_2F_2 (e) T-$ZrSe_2F_2$ (f) T-$HfSe_2F_2$

The band structure of monolayer T-$FeSe_2F_2$ is displayed in Figure S2(a). All bands are spin-degenerate, and the DFT calculations give zero total magnetic moments per unit cell, indicating monolayer T-$FeSe_2F_2$ is a non-magnetic semiconductor. Without considering SOC, the Fermi level does not pass through the valence and conduction bands. The phonon dispersion of monolayer T-$FeSe_2F_2$ is displayed in Figure S2(d). Except for some negligibly small imaginary frequencies near the Γ point due to numerical errors induced by the acoustic sum rule, the absence of imaginary modes throughout the Brillouin zone (BZ) suggests monolayer T- $FeSe_2F_2$ is dynamically stable. The band structures of T-$RuSe_2F_2$ and T-$OsSe_2F_2$, as shown in Figures S2(b) and S2(c), and their stabilities, as depicted in Figures S2(e) and S2(f), are similar.

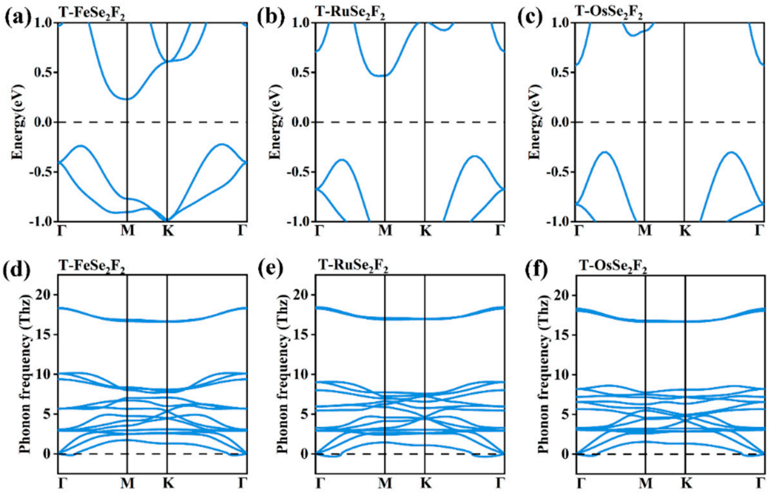

Figure S2. The band structure of monolayer (a) T-$FeSe_2F_2$, (b) T-$RuSe_2F_2$, (c)T-$OsSe_2F_2$.; The phonon spectrum of monolayer (d)T-$FeSe_2F_2$ (e) T-$RuSe_2F_2$ (f)T-$OsSe_2F_2$.

Similar to monolayer T-$FeSe_2F_2$, the band structures of T-RuS_2F_2, T-OsS_2F_2 and T-$OsTe_2F_2$, as shown in Figures S3(a), S3(b) and S3(c), and their stabilities, as depicted in Figures S3(d), S3(e) and S3(f), are similar.

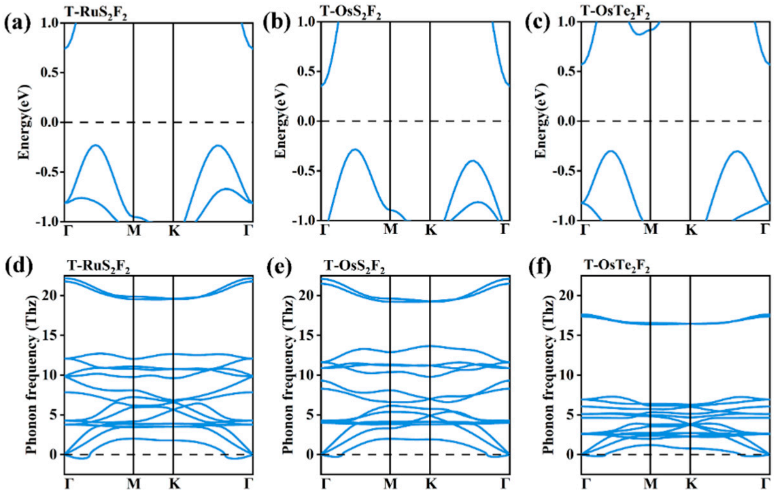

Figure S3. The band structure of monolayer (a) T-RuS_2F_2, (b) T-OsS_2F_2, (c) T-$OsTe_2F_2$.; The phonon spectrum of monolayer (d) T-RuS_2F_2, (e) T-OsS_2F_2, (f) T-$OsTe_2F_2$.

Figure S4(a) displays the band structure of monolayer T-$CrSe_2F_2$ without considering the spin-orbit coupling (SOC) effect. The spin-up bands are gapped while the spin-down bands pass through the Fermi level, suggesting T-$CrSe_2F_2$ is half-metallic. For the spin-down bands, there is only a Weyl point located at the high symmetry K point. The phonon dispersion of monolayer T-$CrSe_2F_2$ is displayed in Figure S4(b).

Except for some negligibly small imaginary frequencies near the Γ point due to numerical errors induced by the acoustic sum rule, the absence of imaginary modes throughout the Brillouin zone (BZ) suggests monolayer T-$CrSe_2F_2$ has dynamic stability.

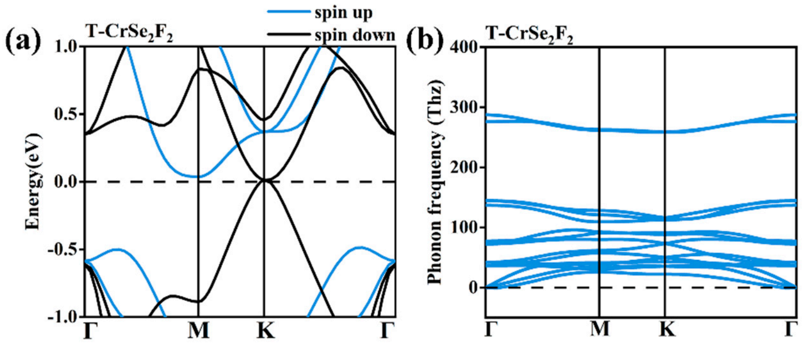

Figure S4. (a)The band structure of monolayer T-$CrSe_2F_2$. (b)The phonon spectrum of monolayer T-$CrSe_2F_2$.

DFT calculations suggest the monolayer T-$CoSe_2F_2$ is a magnetic metal. As shown in Figure S5(a), both the spin-up and spin-down bands cross the Fermi level. Notably, there is only a small portion of one spin-down band that passes through the Fermi level, and there is a big gap below the Fermi level. The phonon dispersion of monolayer T-$CoSe_2F_2$ is displayed in Figure S5(b). Except for some negligibly small imaginary frequencies near the Γ point due to numerical errors induced by the acoustic sum rule, the absence of imaginary modes throughout the Brillouin zone (BZ) suggests monolayer T-$CoSe_2F_2$ has dynamic stability.

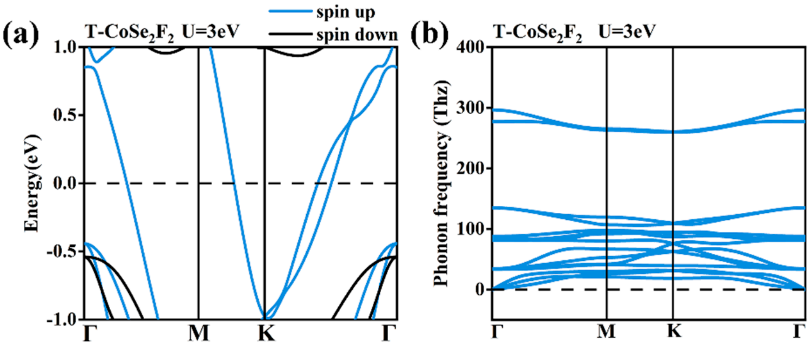

Figure S5. Under U=3 eV, (a) the band structure of monolayer T-$CoSe_2F_2$. and (b) the phonon spectrum of monolayer T-$CoSe_2F_2$.

The phonon dispersion of monolayer T-$CoSe_2F_2$ is displayed in Figure S6(a). Except for some negligibly small imaginary frequencies near the Γ point due to

numerical errors induced by the acoustic sum rule, the absence of imaginary modes throughout the Brillouin zone (BZ) suggests monolayer T-$CoSe_2F_2$ has dynamic stability. Similarly, the phonon spectra of T-CoS_2F_2 and T-FeS_2F_2 are shown in Figure S6(b) and Figure S6(c), respectively.

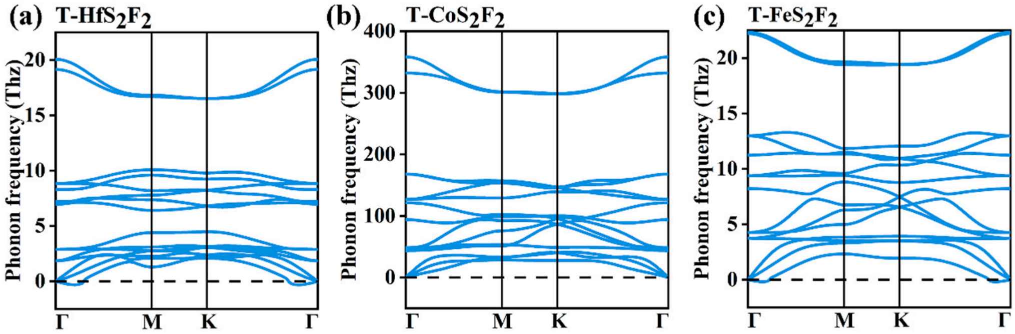

Figure S6. (a) The phonon spectrum of monolayer T-HfS_2F_2; (b) The phonon spectrum of monolayer T-CoS_2F_2. (c)The phonon spectrum of monolayer T-FeS_2F_2.

Table S1. The fluorination formation energy E_f (eV/formula unit) for T-MX_2F_2

	CrS_2F_2	HfS_2F_2	CoS_2F_2	FeS_2F_2
E_f	-3.02	-1.75	-3.43	-3.63

Part II The maximum localized Wannier function fitting results

We have used a perturbation method based on Green's function to investigate the attenuation behavior of magnetic exchange coupling with distance, based on maximally localized Wannier functions (MLWFs), and performed. In this perturbation method, MLWFs are obtained by the wannier90 code based on the DFT results from VASP output. The validity of MLWFs is confirmed by fitting the electronic structure of monolayer T-CrS_2F_2 and T-HfS_2F_2, as shown in Figure S7(a) and Figure S7(b), respectively.

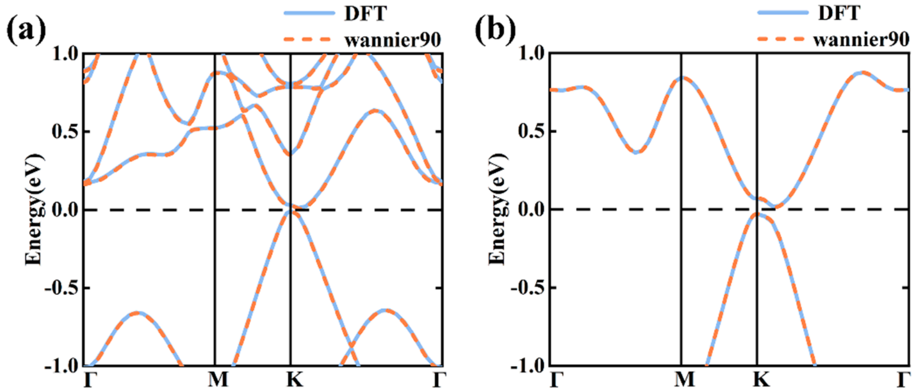

Figure S7. (a) The band structures of monolayer T-CrS_2F_2 obtained by DFT and MLWFs calculations. The Fermi level is set as 0 eV. (b) The band structures of monolayer T-HfS_2F_2 obtained by DFT and MLWFs calculations. The Fermi level is set as 0 eV.

Part III Monte Carlo simulation of Curie temperature

Here, the T_C of monolayer T-CrS_2F_2 is estimated by the classic Monte Carlo method with the following spin Hamiltonian:

$$E = -\sum_{<i,j>} J_{ij}\vec{S}_i \cdot \vec{S}_j - \sum_i A_i |\vec{S}_i^z|^2$$

The exchange parameters J_1 are calculated by the widely used total energy method. We consider exchange interactions for monolayer T-CrS_2F_2: the nest the nearest-neighbor (NN) exchange parameter J_1 for Cr-Cr pairs. The exchange parameters are obtained by calculating the DFT total energies of the two magnetic orders in Figure S8. Based on Table S2, the total energies of the two magnetic orders are given by:

$$E_{FM} = 12J_1S^2 + E_0,$$

$$E_{AFM} = -4J_1S^2 + E_0,$$

$$J_1 = (E_{FM} - E_{AFM})/16S^2,$$

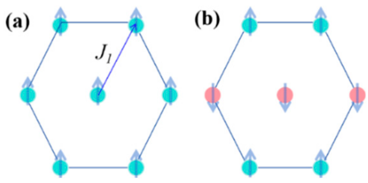

Figure S8. The two magnetic orders (a) FM, (b) AFM.

Table S2. The total energies of two different magnetic order energies in unit of eV/f.u.

E_{FM} (eV)	E_{AFM} (eV)	J_1 (K)	Az (K)
-103.69	-102.96	-525.33	2.03

We use a 30×30×1 supercell to perform the classical Monte Carlo (MC) simulations using the Metropolis algorithm and starting from random spin orders. As shown in Figure S9, the estimated Curie temperature is 577 K for monolayer T-CrS_2F_2, which is far above room temperature and significantly higher than that of $MnBi_2Te_4$ (77 K), a widely studied 2D magnetic topological insulator.

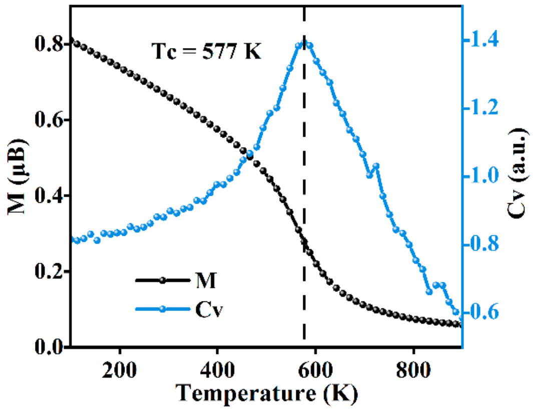

Figure S9. The Curie temperature of T-CrS_2F_2 is determined through Monte Carlo simulations.

Part IV Effect of U on electronic properties and magnetism of T-CoS_2F_2.

For Co-3d electrons, the suitable U values usually are between 1 - 4.0 eV. In this work, we choose the values of 1.0 eV, 2.0 eV, 3.0 eV, and 4.0 eV for Co. To test whether the choice of U will affect the results, we have calculated the band structures of monolayer T-CoS_2F_2 with different U, as shown in Figure. S10. We also calculate the Curie temperature for different U values through Monte Carlo simulation. The larger the U value, the higher the Curie temperature. as shown in Figure. S11.

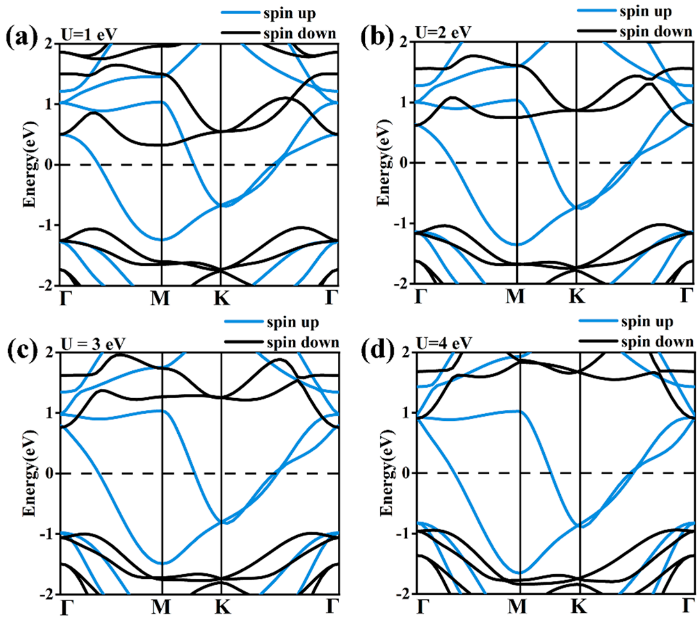

Figure S10. The band structure of monolayer T-CoS_2F_2. (a) U = 1 eV, (b) U = 2 eV, (c) U = 3 eV, (d) U = 4 eV.

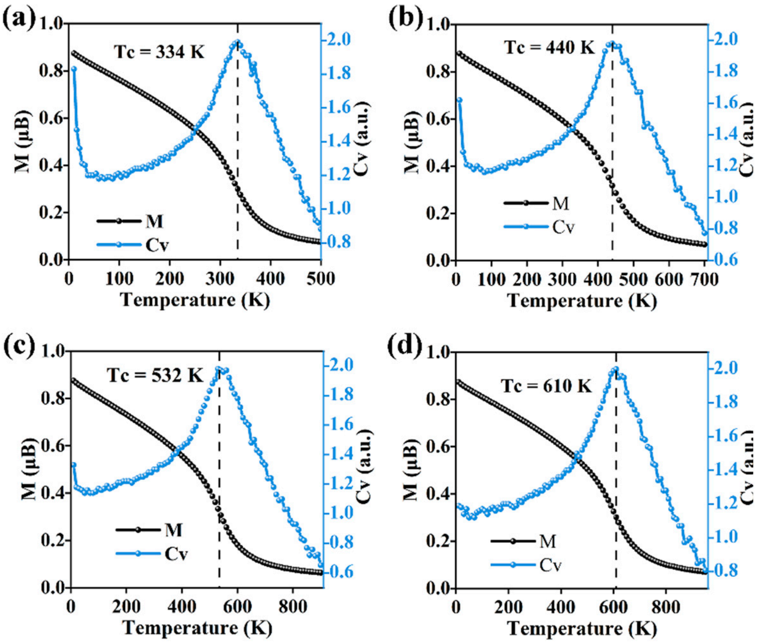

Figure S11. Magnetic moments (*M*) and specific heat (C_V) obtained by MC simulations for T-CoS_2F_2 (a) U = 1 eV, (b) U = 2 eV, (c) U = 3 eV, (d) U = 4 eV.

Part V The HSE06 hybrid functional for the calculation

As shown in the Figure. S12 We use the HSE06 hybrid functional for the calculation. The results showed that T-CrS_2F_2 is a half-semimetal, T-CoS_2F_2 is a half-metal, T-

HfS_2F_2 is a non-magnetic semi-metal, and T-FeS_2F_2 is a non-magnetic semiconductor, which is consistent with the calculation results of the PBE functional. This is to verify the reliability of the calculation conclusion.

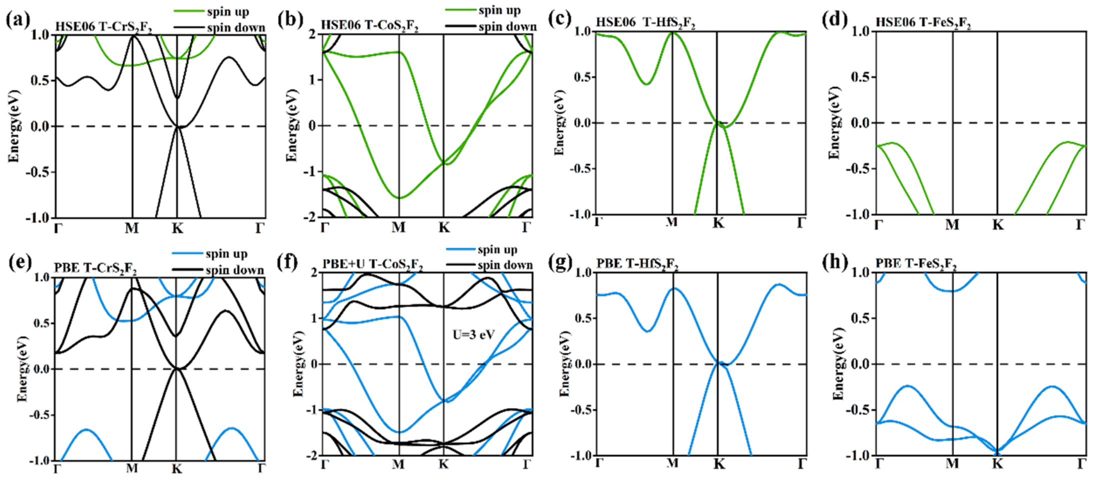

Figure S12. Band structure without considering the SOC effect. Using HSE06 hybrid functional (a) T-CrS_2F_2, (b) T-CoS_2F_2, (c) T-HfS_2F_2, and (d) T-FeS_2F_2. Using PBE functional (e) T-CrS_2F_2, (f) T-CoS_2F_2, (g) T-HfS_2F_2, and (h) T-FeS_2F_2.

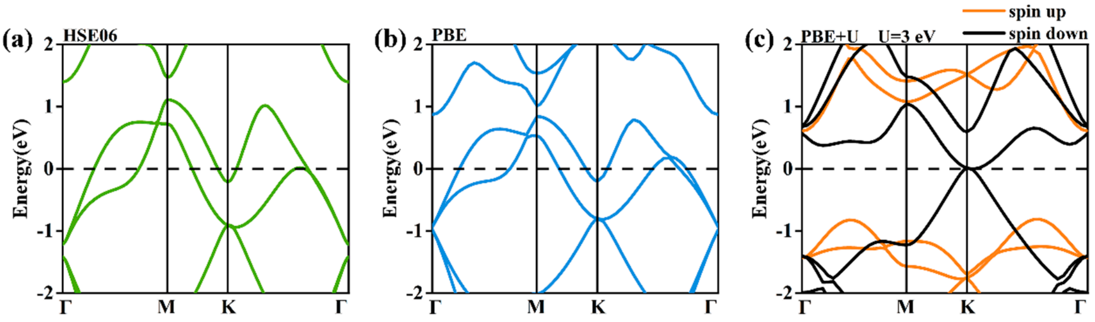

Figure S13. The band structures of monolayer T-MoS_2F_2 under different functionals (a) HSE06 hybrid functional, (b) PBE functional, and (c) PBE+U functional, respectively.

Part VI Electronic and magnetic properties under stress

The band structures of monolayer T-CrS_2F_2 under strain were calculated without considering spin–orbit coupling (SOC) as shown in Figure S14, and with SOC included as presented in Figure S15. The Curie temperature under strain was obtained via Monte Carlo simulations, with the results displayed in Figure S16. Corresponding computational parameters are provided in Table S3. Additionally, the bands structure

of monolayer T-CoS_2F_2 are shown in Figure S17, and its strain-dependent Curie temperature determined from Monte Carlo simulations are given in Figure S18. Corresponding computational parameters are provided in Table S4.

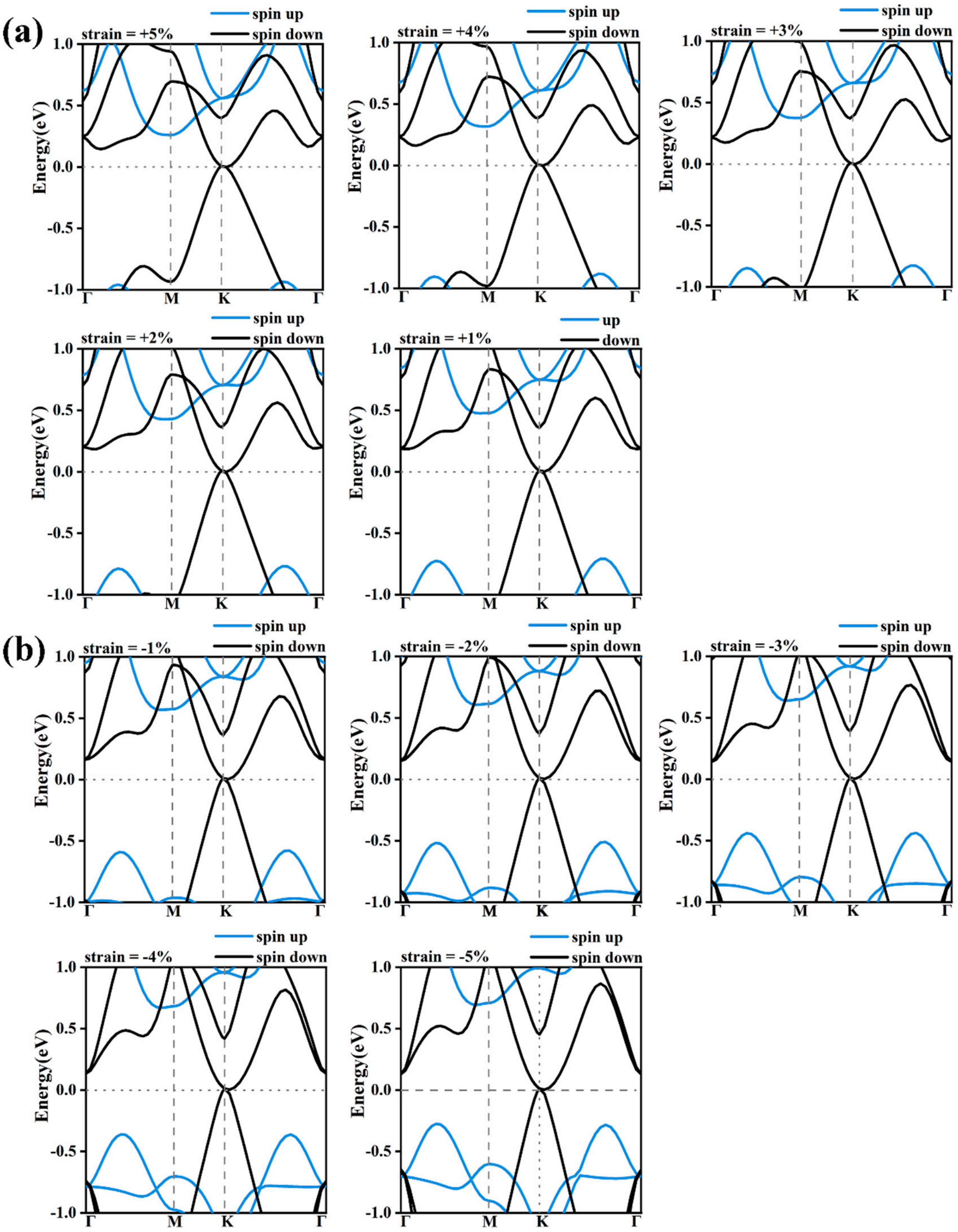

Figure S14. The band structure of monolayer T-CrS_2F_2 under strain without considering SOC. (a) tensile strain; (b) compressive strain.

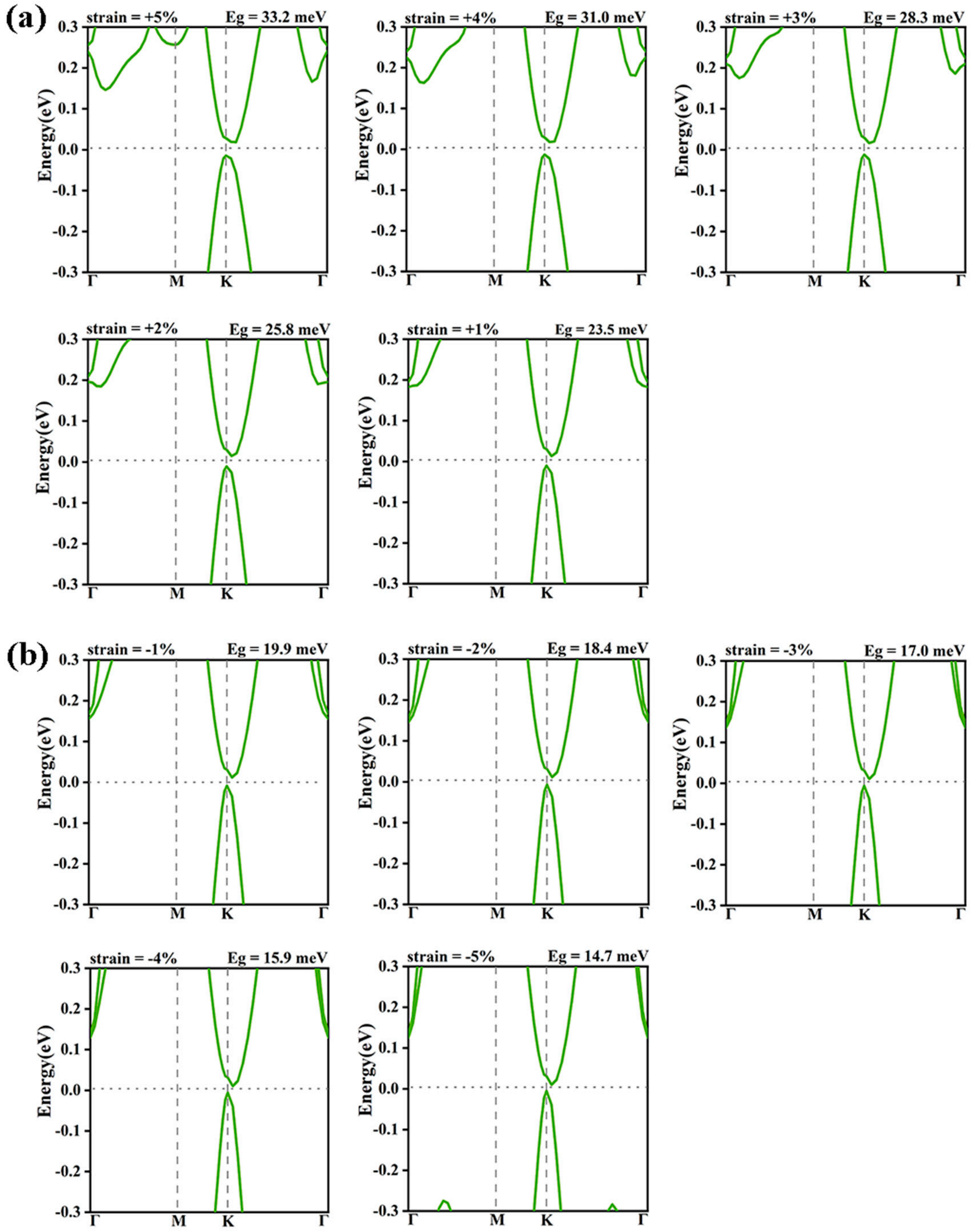

Figure S15. The band structure of monolayer T-CrS_2F_2 under strain considering SOC. (a) tensile strain; (b) compressive strain.

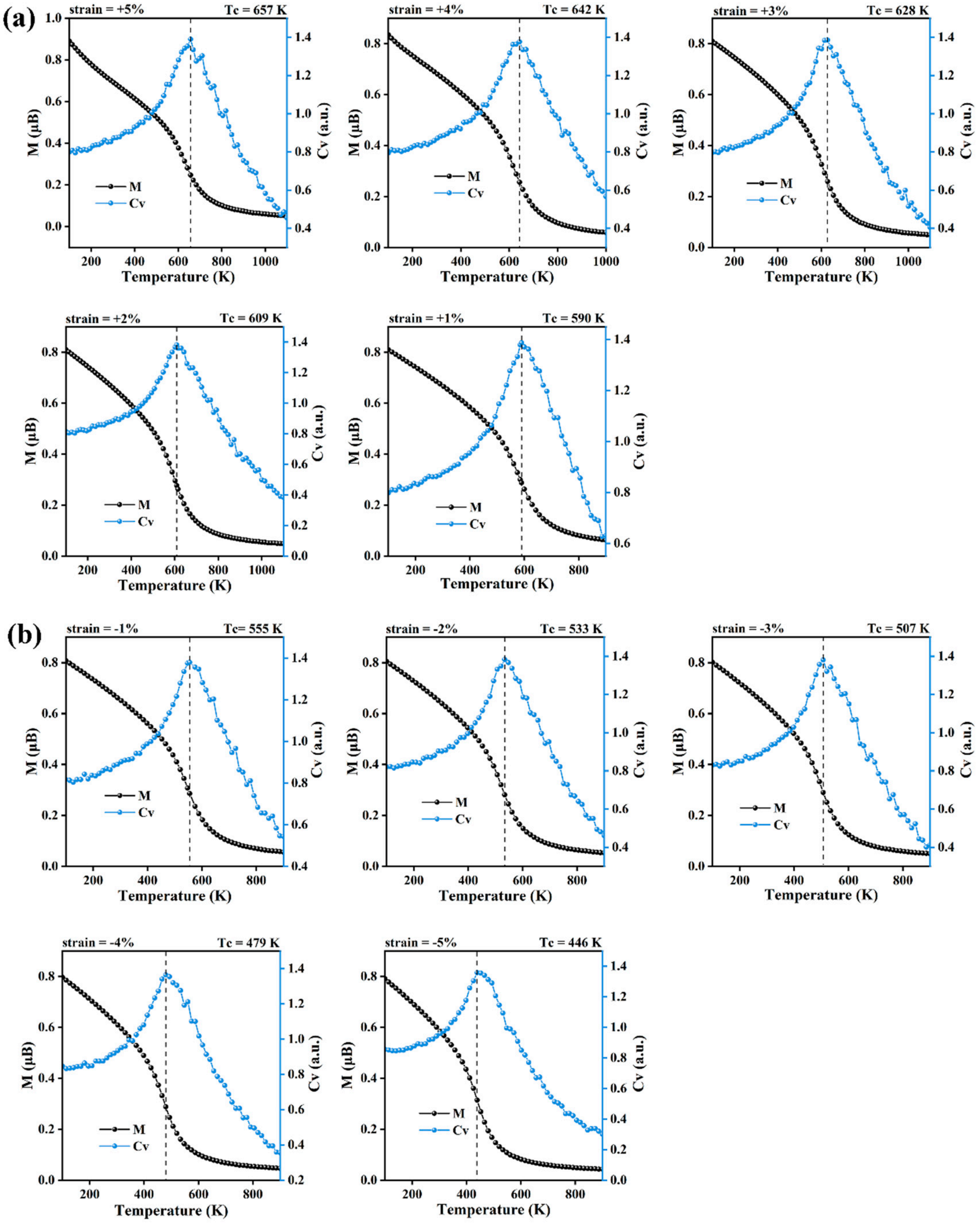

Figure S16. Through Monte Carlo simulation, the Curie temperature of monolayer T-CrS_2F_2 under strain (a) tensile strain; (b) compressive strain

Table S3. The total energy of the ferromagnetic (E_{FM}) and antiferromagnetic (E_{AFM}) states of T-CrS_2F_2, as well as the nearest-neighbor exchange coupling parameter (J_1) and the magnetic anisotropy parameter (A_i).

**T-CrS_2F_2**	**E_{FM}**	**E_{AFM}**	***J_1***	***A_i***
5	-103.1	-102.29	-585.83	0.85
4	-103.3	-102.51	-576.24	0.31
3	-103.47	-102.69	-565.26	-0.19
2	-103.59	-102.82	-554.15	-0.68
1	-103.66	-102.91	-540.4	-1.16
0	-103.68	-102.96	-525.33	-2.03
-1	-103.66	-102.96	-507.91	-2.04
-2	-103.57	-102.9	-487.14	-2.46
-3	-103.43	-102.79	-465.38	-2.85
-4	-103.22	-102.62	-439.6	-3.21
-5	-102.95	-102.39	-409.35	-3.57

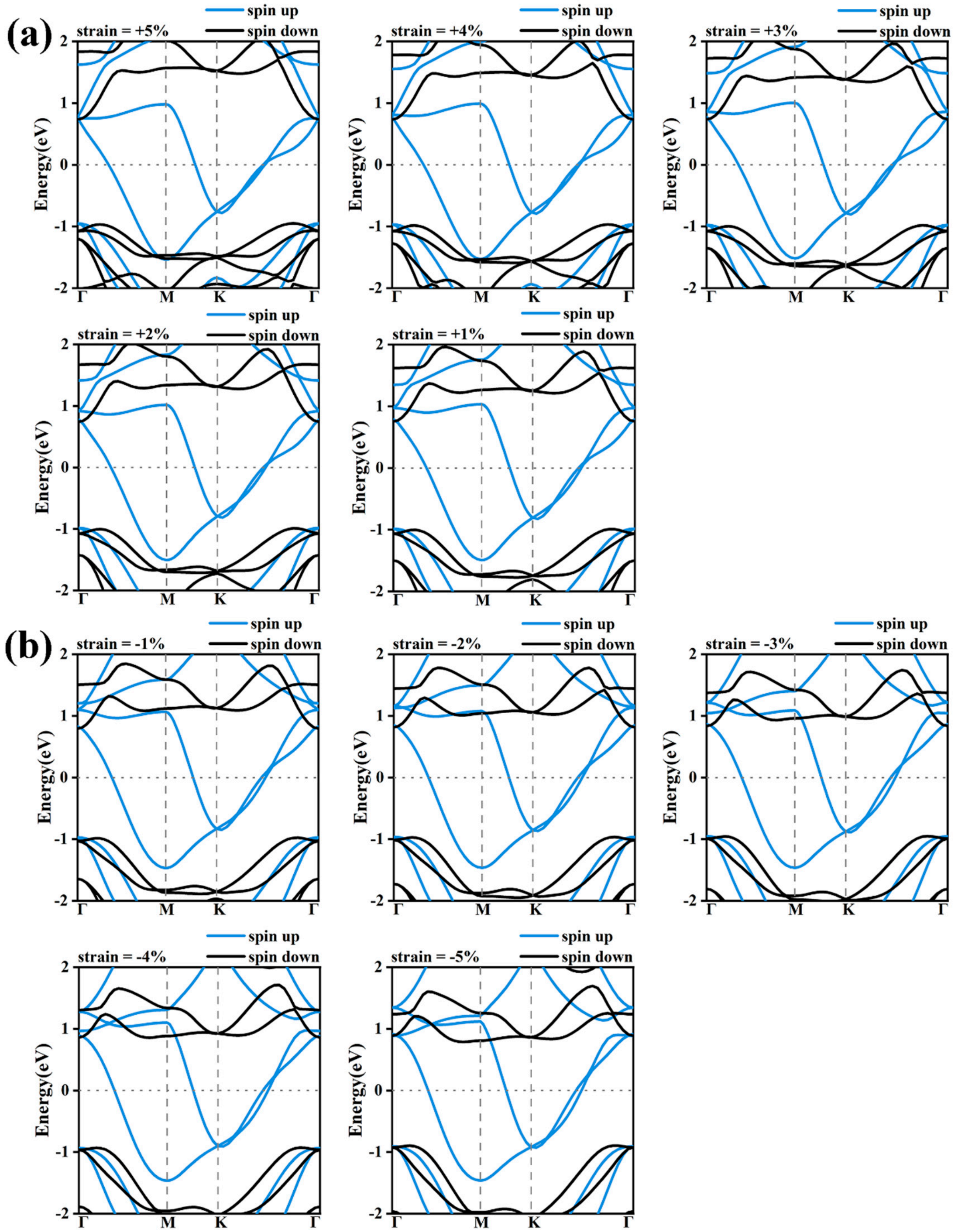

Figure S17. The band structure of monolayer T-CoS_2F_2 under strain. (a) tensile strain; (b) compressive strain.

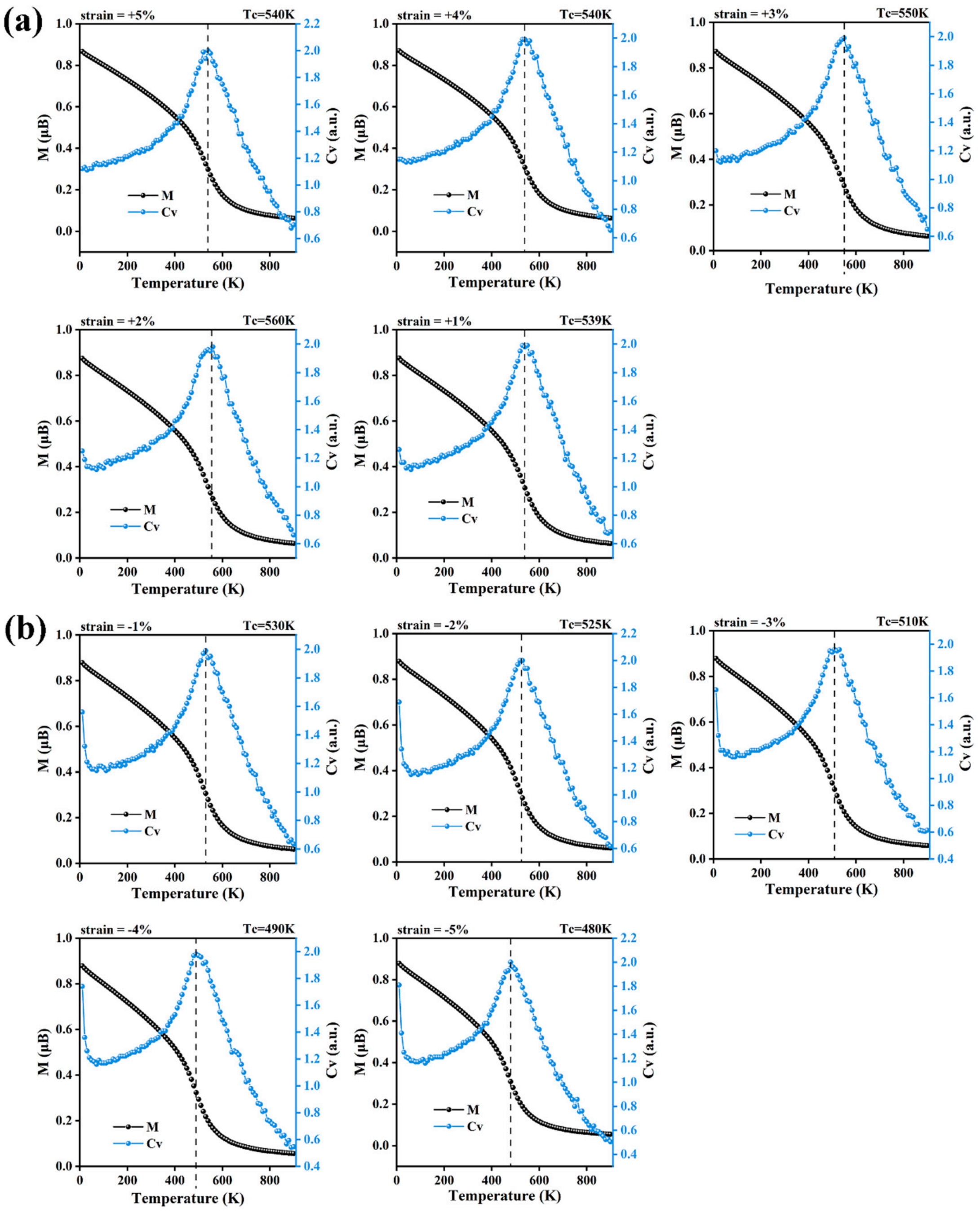

Figure S18. Through Monte Carlo simulation, the Curie temperature of monolayer T-CoS_2F_2 under strain (a) tensile strain; (b) compressive strain

Table S4. The total energy of the ferromagnetic (E_{FM}) and antiferromagnetic (E_{AFM}) states of T-CoS_2F_2, as well as the nearest-neighbor exchange coupling parameter (J_1) and the magnetic anisotropy parameter (A_i).

T-CoS_2F_2	FM	AFM	J_1	A_i
5	-82.68	-82.01	-484.08	-0.14
4	-82.82	-82.15	-487.4	-0.31
3	-82.93	-82.25	-488.58	-0.5
2	-82.99	-82.32	-488.87	-0.7
1	-83.01	-82.34	-487.5	-0.89
0	-83.01	-82.34	-487.17	-0.9
-1	-82.89	-82.23	-477.71	-1.33
-2	-82.74	-82.09	-469.53	-1.59
-3	-82.53	-81.9	-459.41	-1.83
-4	-82.25	-81.64	-447.17	-2.02
-5	-81.9	-81.3	-433.87	-2.15

Part VII T-CrS_2F_2 and T-CoS_2F_2 ferromagnetic ground state

We calculate T-CrS_2F_2 and T-CoS_2F_2 using four different magnetic orders (FM, AFM1, AFM2, AFM3), as shown in Figure S19. The energy of FM is the lowest for both, and they were all in the FM state, as shown in Table S5.

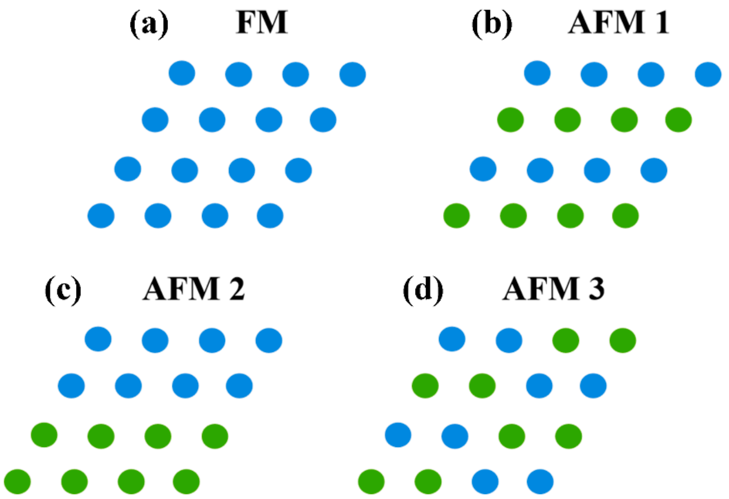

Figure S19. Four different magnetic order configurations: (a) FM (b) AFM1 (c) AFM2 (d) AFM3

Table S5. The total energies of four different magnetic order energies in unit of eV/f.u.

	E_{FM}	$E_{AFM\ 1}$	$E_{AFM\ 2}$	$E_{AFM\ 3}$
Cr	-414.7345	-411.84926	-413.27373	-411.63347
Co	-367.40612	-367.11275	-367.11276	-367.11282

Contrary to conventional expectations that fluorination will change the transition metal atom M in MX_2 from M^{4+} state into M^{6+} state, our differential charge density analysis suggests that the charge transfer after fluorination are mainly occurs between X and F atoms while the charges on M atoms changes much less, as shown in Figure S20 below. This indicates M atoms remain the M^{4+} state after surface fluorination and explains why T-CrS_2F_2 remains 2 μ_B magnetic moments per unit cell.

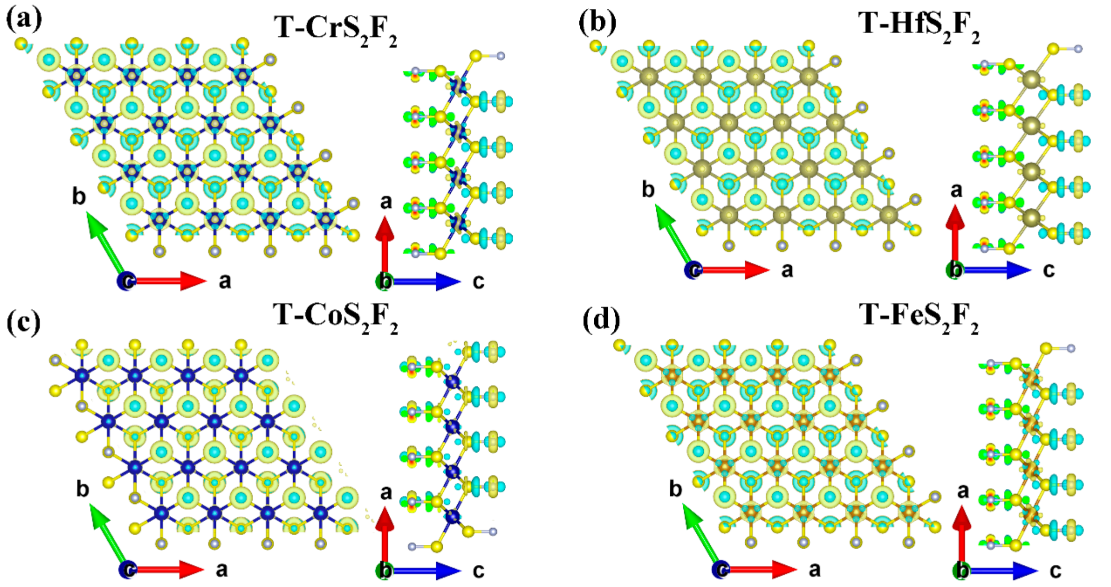

Figure S20. Differential charge density of (a) monolayer T-CrS_2F_2, T-HfS_2F_2, T-CoS_2F_2, and T-FeS_2F_2, respectively. Yellow and cyan represent electron accumulation and loss, respectively.
